# Hidradenitis suppurativa as a potential risk factor of periodontitis: a multi-center, propensity-score-matched cohort study

**DOI:** 10.7150/ijms.93178

**Published:** 2024-03-25

**Authors:** Shuo-Yan Gau, Yu-Chen Guo, Hsin-Yo Lu, Chen-Yu Lin, Chien-Ying Lee, Ru-Yin Tsai, Hui-Chin Chang, Meng-Che Wu, Chen‐Pi Li

**Affiliations:** 1 School of Medicine, Chung Shan Medical University, Taichung, Taiwan.; 2 Department of Medical Education, Kaohsiung Chang Gung Memorial Hospital, Kaohsiung, Taiwan.; 3 Orthopedics Department, Chi-Mei Medical Center, Tainan, Taiwan.; 4 Department of Pharmacy, Chung Shan Medical University Hospital, Taichung, Taiwan.; 5 Department of Pharmacology, Chung Shan Medical University, Taichung, Taiwan.; 6 Department of Anatomy, School of Medicine, Chung Shan Medical University, Taichung, Taiwan; 7 Department of Medical Education, Chung Shan Medical University Hospital, Taichung, Taiwan; 8 Evidence-based Medicine Center, Chung Shan Medical University Hospital, Taichung, Taiwan.; 9 Library, Chung Shan Medical University Hospital, Taichung, Taiwan.; 10 Division of Gastroenterology, Children's Medical Center, Taichung Veterans General Hospital, Taichung, Taiwan.; 11 Department of Post-Baccalaureate Medicine, College of Medicine, National Chung Hsing University, Taichung, Taiwan.; 12 Pediatric Inflammatory Bowel Disease Center, Massachusetts General Hospital for Children, Boston, MA, USA.; 13 Department of Nursing & Tungs' Taichung MetroHarbor Hospital, Taiwan

**Keywords:** hidradenitis suppurativa, periodontitis, cohort, epidemiology, electronic medical records

## Abstract

**Background:** Hidradenitis suppurativa (HS) is a chronic inflammatory skin disease associated with systemic symptoms. Periodontitis, a prevalent dental disease, shares immune-mediated inflammatory characteristics with HS. This cohort study aims to evaluate the association between HS and periodontitis.

**Methods:** Using the TriNetX research network, a global-federated database of electronic health records, we conducted a retrospective cohort study. People being diagnosed of HS were identified and propensity score matching was performed to identify proper control group, via balancing critical covariates Within the follow-up time of 1 year, 3 year and 5 years, hazard ratios were calculated to assess the risk of periodontitis in HS patients compared to controls.

**Results:** Within the 53,968 HS patients and the same number of matched controls, the HS patients exhibited a significantly increased risk of developing periodontitis compared to controls after 3 years of follow-up (HR: 1.64, 95% CI: 1.11, 2.44) and 5 years of follow-up (HR: 1.64, 95% CI: 1.21, 2.24) of follow-up. Sensitivity analyses supported these findings under various matching models and washout periods. While comparing with patients with psoriasis, the association between HS and periodontitis remained significant (HR: 1.73, 95% CI: 1.23, 2.44).

**Conclusion:** The observed increased risk suggests the need for heightened awareness and potential interdisciplinary care for individuals with HS to address periodontal health.

## Introduction

Hidradenitis suppurativa (HS) is a recurrent skin disease with multiple systemic symptoms. It is characterized by chronic inflammatory lesions in intertriginous areas. According to current evidences, the prevalence ranges from 0.00033% to 4.10% in different studies and populations, with a female-predominant gender difference 1. In HS patients, involvement of comorbidities in various organ system were commonly observed 2-5. This may be attributed to the systemic inflammatory nature of HS pathogenesis. The obligatory diagnostic criteria for HS consist of a recurring incidence of painful or purulent wounds occurring more than twice within a six-month period. Clinically, initial lesions like follicular papules or abscesses, along with subsequent lesions such as cysts, fistulas, or sinuses, were also observed 6.

Periodontitis is a prevalent infectious dental disease. Several risk factors contribute to periodontitis, such as low socioeconomic status, smoking, diabetes mellitus, and malnutrition 7. Clinical manifestation of periodontitis includes gingivitis, bleeding, and tooth loss. Estimates of the prevalence of periodontitis vary from clinical classification, and its severe form accounts for 11% globally 8. The diagnostic criteria and grading system for periodontitis were developed to categorize the disease's severity and extent based on clinical attachment loss, radiographic bone loss, and tooth loss resulting from periodontitis. The grading system, ranging from Grade A to C, is designed to indicate the speed of disease progression, response to standard therapy, and potential impact on systemic health, considering risk factors such as smoking and diabetes 9.

Both innate immunity and adaptive immunity are involved in the pathogenesis of periodontitis. Dysregulation of neutrophils, B lymphocytes, T lymphocytes, antigen-presenting cells, and complement leads to a persistent inflammatory condition, causing the growth and maintenance of the dysbiotic microbial community.

A cross-sectional study by Jastrząb et al shed light on the similarities of HS and periodontitis. Compared to health controls, HS patients tend to be infected with perio-pathogenic genera of bacteria 10. Recent studies also demonstrate that Th17/IL-17 and IL-23 play a crucial role in periodontitis and other immune-mediated inflammatory diseases 11. These findings indicate that periodontitis and HS may share a similar oral microbiome composition, with a comparable immunological pathway.

Previous studies have indicated a strong association between HS and immune-mediated diseases 12. HS and periodontitis are both considered immune-mediated inflammatory diseases, with highly similar immune system responses. However the long-term relationship between HS and periodontitis remains unclear to date. Therefore, we conducted a cohort study to provide robust evidence between HS and periodontitis.

## Methods and Materials

### Data source and study design

We performed a retrospective cohort study to evaluate the association between HS and periodontitis. Data was retrieved from the TriNetX research network, a global-federated research database with de-identified electronic health records (EHR) provided by more than 120 collaborative healthcare organizations (HCOs), and the database has been widely applied in various specialties in the research of clinical and experimental medicine 13, 14. The TriNetX research network provided accesses to the subsets consists of HCOs in different regions, including the United States, Europe and Asia. For our study, we focused on the US collaborative network, which provides EHR data from HCOs in the United States. This subset has been previously employed in recent publications assessing HS comorbidities 15, 16.

### Study population and outcome evaluation

During the study period from January 1st, 2005, to December 31st, 2017, we gathered data for the case cohort by selecting patients with visit records to healthcare organizations (HCOs) and a diagnosis of HS. The non-HS control cohort comprised individuals who underwent health examinations and had visit records to HCOs. Exclusions from both cohorts included individuals who died before the index date, had previous periodontitis or cancer records, or were under 18 years old. Data in the TriNetX research network, including visit records, diagnoses, medication records, lab data, and procedures, were available for analysis. Detailed algorithms for disease and medication definitions are presented in **Table S1**. After excluding ineligible participants, 1:1 propensity score matching was performed based on covariates influencing incident outcome events. Following matching, we enrolled 53,968 HS patients and an equal number of controls for further comparisons. The outcome event was defined as the occurrence of periodontitis. In the main analysis, each patient was followed up for 1 year, 3 years, and 5 years to determine the risk of the outcome event in the HS cohort. Given that the hazard ratio is designed to analyze time-to-event data, we set cutoff points for different follow-up periods to more clearly evaluate whether differences in follow-up times could affect the observed association between HS and periodontitis. Incident periodontitis occurring within 3 months after the index date was excluded from the main analysis.

### Stratifications and Sensitivity analysis

Stratification analyses were performed based on different age groups (18-64 years old and greater than 65 years old) and sex (male/female) to assess the detailed status of the HS-periodontitis association. Moreover, in addition to age and sex, considering that the status of periodontitis is associated with sex hormones, we also stratified the analysis based on menopausal status. To validate the findings, sensitivity analyses were conducted using multiple matching algorithms and various wash-out periods to address potential overmatching bias and reversed causality. Additionally, recognizing the association between psoriasis and a high risk of periodontitis 17, we included psoriasis patients as an active comparator. This allowed us to evaluate the risk of periodontitis in HS patients in comparison to individuals with psoriasis.

### Statistical analysis

All formal analyses were conducted on September 9th, 2023, using the analytical system within the TriNetX research network. In each analysis, the hazard ratio (HR) was calculated to assess the future risk of periodontitis in HS patients. The significance of the calculated HR was determined by applying a 95% confidence interval (95% CI). When presenting baseline information for study participants, the standardized difference (SD) was utilized to assess whether baseline characteristics differed between the two cohorts before and after matching. A SD value smaller than 0.1 indicated that the difference between the two groups was statistically insignificant. Regarding the implementation of propensity score matching, the greedy nearest neighbor algorithms were utilized, employing a caliper width of 0.1.

### Ethical Issues

The Institutional Review Board of Tungs' Taichung MetroHarbor Hospital exempted the need of patient consent in this study (IRB TTMHH No.:112208N).

## Results

Before matching, there were 53,971 HS patients and 5,104,067 controls enrolled in the study. Initially, there were notable disparities in baseline covariates, encompassing age, sex, lifestyle, and comorbidity status, between the two groups. However, following the matching process, significant distinctions in confounding variables, such as age, sex, race, socioeconomic status, lifestyle habits, comorbidity status, medical utilization, and laboratory data, were not evident between the two groups (**Table 1**). Among the HS patients, the average age stood at 33.8 years, predominantly consisting of females (74.4%). The racial composition indicated that 44.5% were White, 35.3% Black or African American, 1.6% Asian, and 0.6% American Indian or Alaska Native.

**Figure 2:**
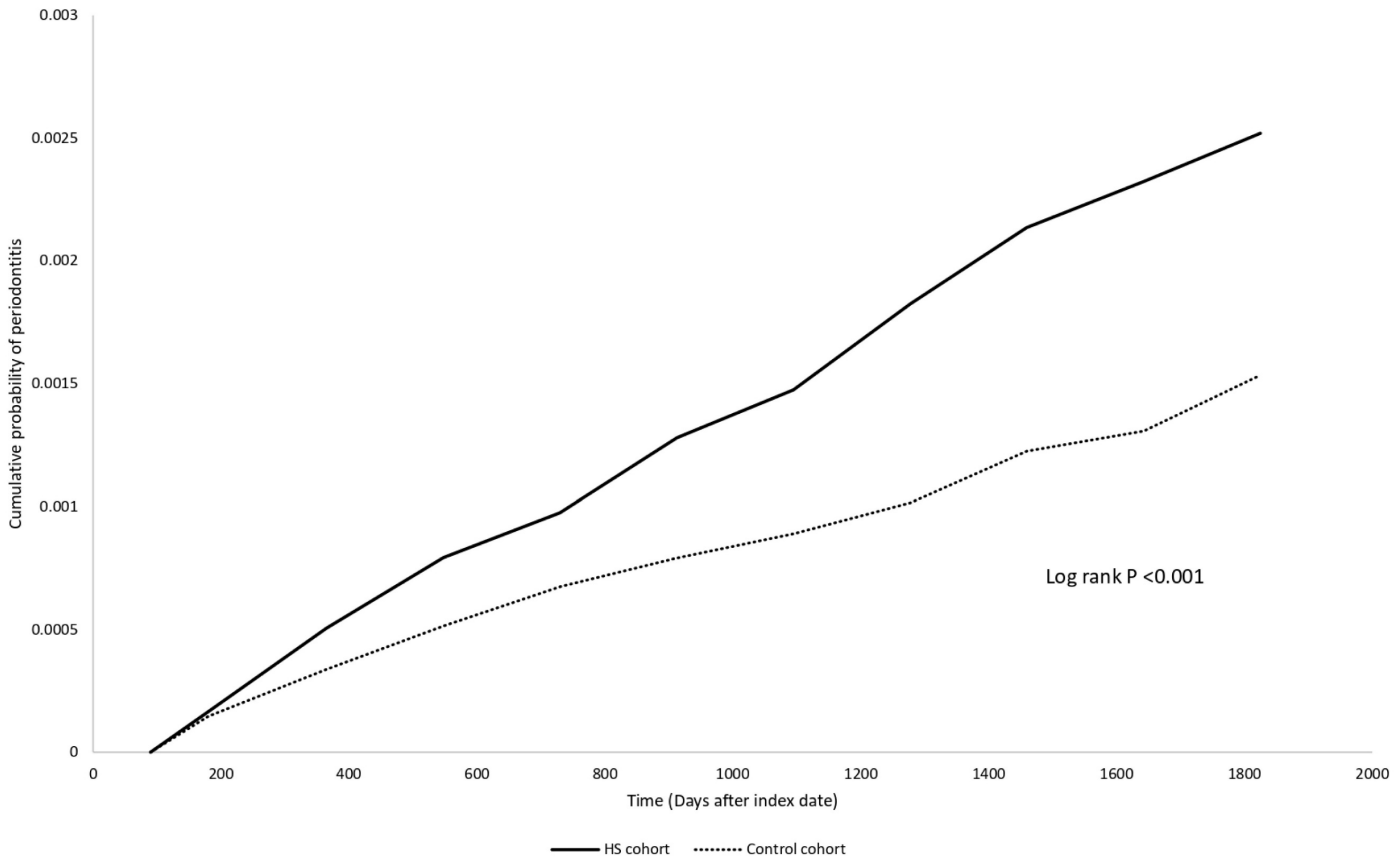
Kaplan-Meier plot

**Table 2** unveils the risk for HS patients developing periodontitis, compared to the control group. The HR was found to be 1.50 (95% CI: 0.80, 2.82) after one year of follow-up, 1.64 (95% CI: 1.11, 2.44) after three years, and 1.64 (95% CI: 1.21, 2.24) after five years. The Kaplan Meier curve was presented in **Figure 2**. Sensitivity analyses under different matching models and applied washout periods, as detailed in **Tables S2-S3**, consistently support this finding in 5-year follow-up period. Intriguingly, over the 5-year follow-up period, we observed that people with HS displayed a greater risk for developing periodontitis compared to patients with psoriasis (HR: 1.73, 95% CI: 1.23, 2.44) (**Table S4**).

The stratification analysis reveals that males exhibit a hazard ratio (HR) of 2.12 (95% CI: 1.23, 3.66), females have an HR of 1.51 (95% CI: 1.03, 2.22), and individuals aged between 18-64 possess an HR of 1.67 (95% CI: 1.21, 2.31). Notably, all different subgroups of HS patients, categorized by sex and age (18 up to 64 years), significantly face a heightened risk of developing periodontitis compared to the control group. However, we were unable to calculate the risk of periodontitis development in HS patients due to the limited number of patients presenting with incident periodontitis (**Table 3**).

## Discussion

We report an increased short-term risk for HS patients developing periodontitis, with a hazard ratio of 1.64 (95% CI: 1.21, 2.24) compared to non-HS controls. This observed association applies across different age and sex subgroups; however, the risk of periodontitis in older HS patients (greater than 65 years old) could not be evaluated.

Some studies have highlighted a potential relationship between hidradenitis suppurativa and periodontal disease. A recent cross-sectional study demonstrated a high prevalence of periodontitis in HS patients compared to healthy controls 18. However, unlike most existing data, our study provides evidence of the longitudinal association between the two diseases.

The etiology of HS is multifaceted, involving genetics, hormonal factors, innate immunity, and environmental influences 19. HS has strong association with various inflammatory comorbidities, including cardiometabolic disorders (e.g., diabetes mellitus, hyperlipidemia) and endocrine conditions (e.g., thyroid dysfunction, polycystic ovarian syndrome) 20-22.

Multiple pathways including IL-23 and IL-12/TH1 pathway has been identified to play roles in the immunological status of HS patients 23. A feed-forward inflammation mechanism of T-helper cell 17 (TH17) has been recognized in the self-perpetuating clinical disease 24. While the exact mechanisms of TH17 feed-forward self-amplification in HS remain unclear, it is hypothesized that TH17 immune responses may occur more frequently in apocrine-gland-rich areas, similar to the activation seen in psoriasis 25.

Epithelial hyperplasia and keratinization of hair follicles lead to follicular occlusion 26, 27. Chemokine gradients (CXCL1/CXCL8) created by keratinocytes in obstructed follicles attract inflammatory cells 28. The interaction between activated keratinocytes, inflammatory and stromal cells activates TH1, TH17, fibroblasts, dendritic cells, and neutrophils through various pathways 29, 30. Cytokines in the IL-17 family, which are triggered by TH17, promotes the release of IL-1β, IL-6, and TNF-α, triggering a complement-mediated inflammatory response. Neutrophils migrate into the skin more efficiently facilitated by IL-1β 31, while IL-6 enhances the proliferation ability of keratinocytes 32. TNF-α, IL-1, and IL-6 activate dendritic cells, inducing the secretion of IL-12 and IL-23 to help the maturation of TH1 and TH17, leading to the amplification of inflammatory responses 33. This establishes the circulation of chronic inflammation in HS.

Prior studies have documented a connection between inflammatory skin diseases and periodontal conditions. Specifically, certain research has indicated a correlation between psoriasis and periodontitis, with the strength of this association escalating in tandem with the severity of psoriasis 34, 35. In the pathogenesis of periodontitis, the inflammatory involvement of TH17 and the IL-23/IL-17 axis has been recognized 11. IL-23, an essential cytokine, plays a crucial role in differentiating and expanding the TH17 subset. In periodontal lesions, significantly higher levels of IL-23 have been detected compared to healthy controls 36, 37. In periodontitis patients, the IL-17 family were deemed as cytokines exhibiting potent pro-osteoclastogenic capability, potentially contributing to the development of periodontitis 38. It may also stimulate the synthesis of matrix metalloproteinase in epithelial cells, endothelial cells, and fibroblasts 39. HS and periodontitis share highly overlapping immune pathways, specifically involving the dysregulated TH17 pathway. Given that IL-17 is a crucial cytokine in the pathogenesis of both HS and periodontitis, dysregulated secretion might contribute to the progression of both diseases. Moreover, the influence on oral microbiome could also potentially be involved in the HS-periodontitis association. Though studies evaluating the changes of oral microbiome in HS patients were scarce 40, some recent clinical studies reported that subgingival microbiome composition in individuals with HS exhibits certain similarities to that of patients with periodontitis^10^, ^18^. It was reported that specific bacteria genera, such as P. gingivalis, T. denticola, T. forsythia, P. micros, F. nucleatum, and C. gingivalis, are implicated in the pathogenesis of periodontal disease and could potentially influence HS progression 10. When self-tolerance mechanisms fail, bacteria may trigger exaggerated inflammatory responses, affecting dendritic cells and Toll-like receptors expressions 36. In this study, although we observed an increased risk of developing periodontitis in HS patients, we were unable to evaluate the actual oral microbiome status of each participant. Due to remaining gaps in understanding the unexplored mechanisms of microorganic, immunological, and genetic factors contributing to the pathogenesis of both conditions, additional extensive studies are essential to enhance the robustness of these research discoveries.

There are several limitations to note in our study. Firstly, notwithstanding our attempts at matching covariates, our research cohort predominantly consists of individuals belonging to White and African ethnic groups, with a lesser representation of Asian and Native American populations. Considering the possible differences in clinical disease patterns across diverse racial groups, the applicability of our results should be approached with caution. Secondly, our study is observational, and as such, the establishment of causation between HS and periodontal disease is not possible. Thirdly, despite sensitivity analyses and confounder matching, the presence of confounding bias and misclassification bias, including unaccounted potential confounders or the possibility of misdiagnosed HS, should be acknowledged. Therefore, the results should be interpreted with caution. Fourthly, in the system of TriNetX research network, information of inflammatory factors such as serum concentration were not available. In this case, we were not able to perform additional analysis to evaluate the status of inflammatory factors in both HS and periodontitis patients. However, in order to further evaluate whether the status of inflammatory status could influence the observed HS-periodontitis association, we performed sensitivity analyses based on adding anti-inflammatory agents, including TNF alpha inhibitors, IL-17 inhibitors and JAK inhibitors as matching covariates (**Table S2, Models 4-6**). In the sensitivity analyses, the existing link between HS and periodontitis persisted. Given that the current study could not provide further evidence on the cytokine-level changes in HS patients, readers should prudently interpret the influence of inflammatory factors in the association between the two diseases.

Utilizing a robust global-federated database, we report real-world evidences of HS as a risk factor for periodontal disease while indicating a possible association between two diseases. This discovery could potentially contribute to increasing awareness regarding this clinical condition.

## Supplementary Material

Supplementary tables.

## Figures and Tables

**Figure 1:**
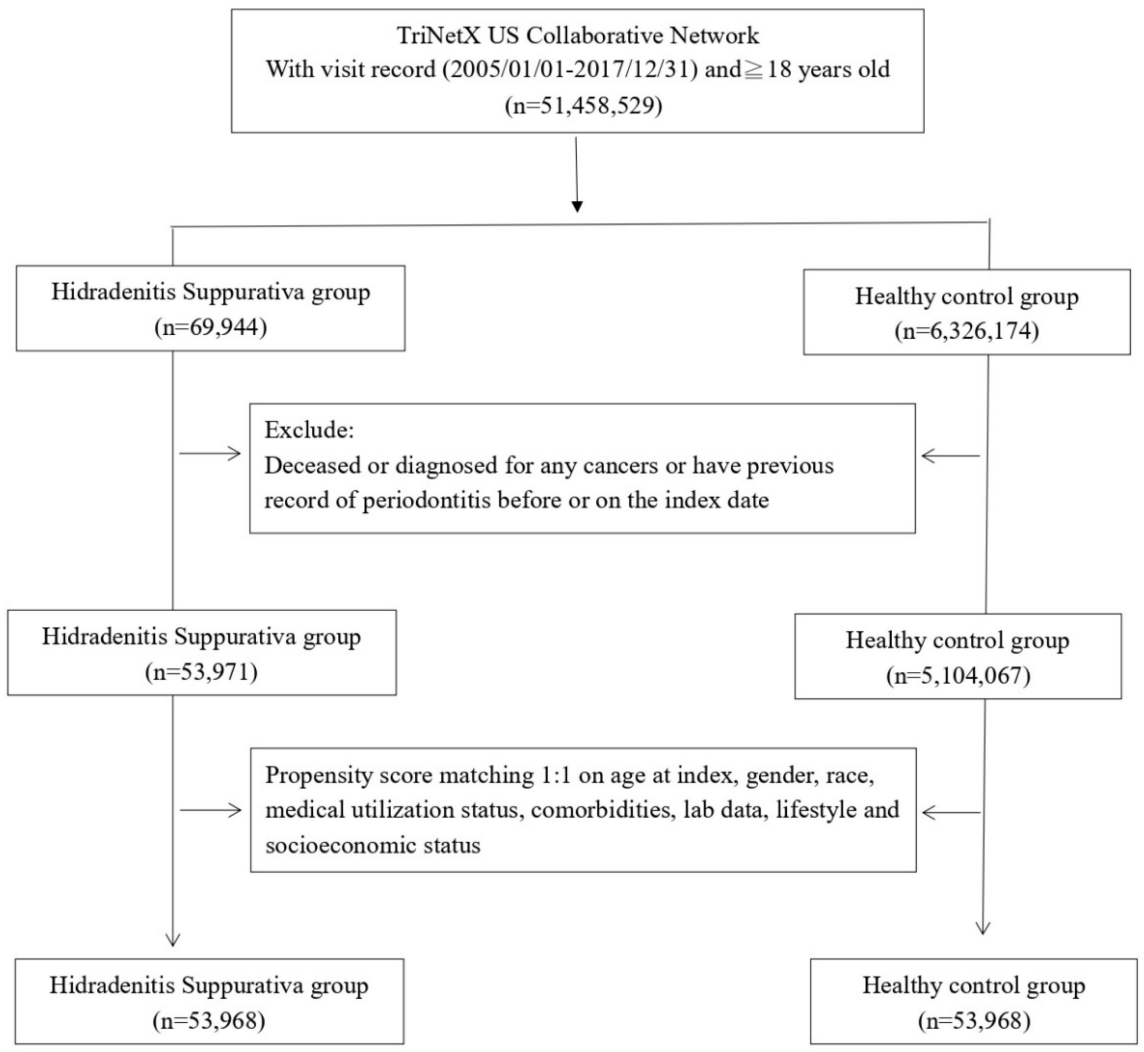
Participant selection flowchart

**Table 1:** Baseline characteristics of study subjects (before and after propensity score matching)

	Before matching			After matching^a^		
	HS cohort (n=53,971)	Control cohort (n= 5,104,067)	Std diff	HS cohort (n=53,968)	Control cohort (n=53,968)	Std diff
**Age at index**						
Mean±SD	33.8±14.2	38.3±20.9	**0.25**	33.8±14.2	33.9±14.3	0.01
**Sex**						
Male	13575(25.2)	2203828(43.2)	**0.39**	13575(25.2)	13575(25.2)	0.00
Female	40177(74.4)	2819228(55.2)	**0.41**	40174(74.4)	40183(74.5)	0.00
**Race, n (%)**						
White	24003(44.5)	3083614(60.4)	**0.32**	24003(44.5)	23954(44.4)	0.00
Black or African American	19040(35.3)	773906(15.2)	**0.48**	19037(35.3)	19193(35.6)	0.01
Asian	855(1.6)	175184(3.4)	**0.12**	855(1.6)	832(1.5)	0.00
American Indian or Alaska Native	227(0.4)	15100(0.3)	0.02	227(0.4)	186(0.3)	0.01
**Socioeconomic status**						
Socioeconomic/psychosocial circumstances problem	1048(1.9)	35990(0.7)	**0.11**	1046(1.9)	1017(1.9)	0.00
**Lifestyle**						
Alcohol dependence, smoking and substance use	6428(11.9)	166399(3.3)	**0.33**	6425(11.9)	6451(12.0)	0.00
**Comorbidities**						
Hypertension	6975(12.9)	517385(10.1)	0.09	6973(12.9)	6931(12.8)	0.00
Diabetes mellitus	4153(7.7)	219179(4.3)	**0.14**	4151(7.7)	4021(7.5)	0.01
Gingivitis	78(0.1)	2488(0.0)	0.03	78(0.1)	49(0.1)	0.02
**Medical Utilization Status**						
Ambulatory visit	33311(61.7)	2480746(48.6)	**0.27**	33308(61.7)	33335(61.8)	0.00
Inpatient visit	10049(18.6)	591513(11.6)	**0.20**	10048(18.6)	10080(18.7)	0.00
**Laboratory data**						
BMI, n (%)						
≧ 35 (kg/m^2^)	4340(8.0)	108365(2.1)	**0.27**	4337(8.0)	4337(8.0)	0.00
C reactive protein, n (%)						
≧ 3 (mg/L)	2203(4.1)	75074(1.5)	**0.16**	2200(4.1)	2152(4.0)	0.00

If the patient is less or equal to 10, results show the count as 10 Bold font represents a standardized difference was more than 0.1 HS: Hidradenitis Suppurativa; ^a^ Propensity score matching was performed on age at index, sex, race, body mass index, status of comorbidities (including diabetes mellitus, hypertension, hyperlipidemia, gingivitis), substance use status, medical utilization status, lab data and socioeconomic status.

**Table 2:** Risk of periodontitis under different follow-up time^a^

Outcomes	Hazard ratio (95% Confidence interval)^b^		
	1 year	3 years	5 years
Periodontitis	1.50 (0.80,2.82)	**1.64 (1.11,2.44)**	**1.64 (1.21,2.24)**

HS: hidradenitis suppurativa ^a^Data present here were the value of follow up from 90 days after index date to the respective following up years. ^b^ Propensity score matching was performed on age at index, sex, race, body mass index, status of comorbidities (including diabetes mellitus, hypertension, hyperlipidemia, gingivitis), substance use status, medical utilization status, lab data and socioeconomic status.

**Table 3:** Stratification analysis of periodontitis risk in HS patients

	Cases occurring new-onset periodontitis		
Subgroups	HS cohort (No. of event/HS patient amount in each subgroup)	Control cohort (No. of event/non-HS patient amount in each subgroup)	HR (95% CI)^a^
**Gender**			
Male	40/13,573	19/13,573	**2.12 (1.23,3.66)**
Female	66/13,573	43/40,175	**1.51 (1.03,2.22)**
**Age at index date**			
18-64 years old	98/48,884	58/48,884	**1.67 (1.21,2.31)**
≥ 65 years old^b^	10/5083	10/5083	**NA**
**Menopause**			
Yes^b^	10/1991	10/1991	**NA**
No	65/39903	35/39903	**1.83 (1.22,2.76)**

^a^ Propensity score matching was performed on age at index, sex, race, body mass index, status of comorbidities (including diabetes mellitus, hypertension, hyperlipidemia, gingivitis), substance use status, medical utilization status, lab data and socioeconomic status. ^b^ In order to protect the privacy of participants, the TriNetX system was not able to present the exact number of participant if the number was less than 10. Hence for these stratification groups, we were not able to calculate the hazard ratio.
