# Supplementary material for: Hidradenitis suppurativa as a potential risk factor of periodontitis: a multi-center, propensity-score-matched cohort study
Source: Int J Med Sci. 2024 Mar 25;21(5):874–81. doi: 10.7150/ijms.93178 (PMC11008474; doi:10.7150/ijms.93178)
Supplement: Supplementary file 1 — Supplementary tables. [file ijmsv21p0874s1.pdf]

Supplementary Files

Supplementary Tables

Table S1. Utilized administrative codes in the current study

**Description**	**ICD-10-CM codes[a]**
Hidradenitis Suppurativa	L73.2
Neoplasms	C00-D49
Periodontitis	K05.3
Gingivitis	K05.1
Diabetes mellitus	E08-E13
Hypertension	I10
Socioeconomic and psychosocial circumstances	Z55-Z65
Substance abuse	F10-F19
Menopause	N95
Encounter for health examination	Z00
**Medications**	**RxNorm/ATC codes**
Fluoxetine	RxNorm:4493
Venlafaxine	RxNorm:39786
Nifedipine	RxNorm:7417
Amlodipine	RxNorm:17767
Mycophenolate	RxNorm:265323
Tacrolimus	RxNorm:42316
Tumor necrotizing factor alpha inhibitors	ATC code: L04AB

Secukinumab	RxNorm:1599788
Brodalumab	RxNorm: 1872251
Tofacitinib	RxNorm:1357536
Baricitinib	RxNorm: 2047232

[a]ICD-10-CM: International Classification of Diseases, Tenth Revision, Clinical Modification

Table S2. Sensitivity analysis: risk of periodontitis in HS patients based on different covariate matching models, with 5-year follow up

Outcomes	Hazard ratio (95% Confidence interval)						
	Crude	Model 1[a]	Model 2[b]	Model 3[c]	Model 4[d]	Model 5[e]	Model 6[f]
Periodontitis	**3.24 (2.67,3.94)**	**2.76 (1.89,4.03)**	**3.14 (2.12,4.66)**	**1.62 (1.19,2.21)**	**1.78 (1.30,2.43)**	**1.70 (1.25,2.31)**	**1.67 (1.23,2.27)**

[a] Propensity score matching was performed on age at index and sex

[b] Propensity score matching was performed on age at index and sex, socioeconomic status and substance use status

[c] Propensity score matching was performed on age at index, sex, race, body mass index, status of comorbidities (including diabetes mellitus, hypertension, hyperlipidemia, gingivitis), substance use status, medical utilization status, lab data and socioeconomic status and comedication status (antidepressants, calcium channel blockers and immunosuppressants)

[d] Propensity score matching was performed based on the same matching covariates as the main analysis in Table 2 + the use of TNF alpha inhibitors

[e] Propensity score matching was performed based on the same matching covariates as the main analysis in Table 2 + the use of Interleukin 17 inhibitors (secukinumab, brodalumab)

[f] Propensity score matching was performed based on the same matching covariates as the main analysis in Table 2 + the use of Janus kinase inhibitors (tofacitinib, baricitinib)

Table S3. Sensitivity analysis: risk of periodontitis with different wash-out periods [a]

Outcomes	Hazard ratio (95% Confidence interval)[b]		
	6 months	12 months	18 months
Periodontitis	**1.71 (1.24,2.36)**	**1.65 (1.17,2.34)**	**1.70 (1.17,2.48)**

[a] Incident events occurred within each wash-out period were excluded in the corresponding analysis, following up for 5 years after index date

[b] Propensity score matching was performed on age at index, sex, race, body mass index, status of comorbidities (including diabetes mellitus, hypertension, hyperlipidemia, gingivitis), substance use status, medical utilization status, lab data and socioeconomic status.

Table S4. Risk of periodontitis in HS patients, comparing with patients with psoriasis in 5-year follow up

Outcomes	HS cohort (No. of event/ HS patient amount in each subgroup)	Psoriasis cohort (No. of event/ Psoriasis patient amount in each subgroup)	Hazard ratio (95% Confidence interval) [a,b]
Periodontitis	89/43,226	51/43,226	**1.73 (1.23,2.44)**

[a] Data present here were the value of follow up from 90 days after index date to the respective following up years.

[b] Propensity score matching was performed on age at index, sex, race, body mass index, status of comorbidities (including diabetes mellitus, hypertension, hyperlipidemia, gingivitis), substance use status, medical utilization status, lab data and socioeconomic status.
